# Member announcements

**DOI:** 10.3109/03009734.2012.709036

**Published:** 2012-08

Dear Members,

There are two forthcoming activities that we would like to announce this time. The first one is our annual meeting with an accompanying dinner that will take place on Wednesday, September 26, 2012 at 5.00 p.m. Further details will be sent out to all members in the beginning of September. The second event is “Rudbeckdagen” that this year will be arranged – as usual in collaboration with our Medical and Pharmaceutical Faculties – on Friday, October 19. This year´s Rudbeck prize winner is professor Bengt Westermark. Again, further details will be sent out in due time.

Torbjörn Karlsson

Anna Rask-Andersen

